# Determining the Hot Workability and Microstructural Evolution of an Fe-Cr-Mo-Mn Steel Using 3D Processing Maps

**DOI:** 10.3390/ma17112715

**Published:** 2024-06-03

**Authors:** Cunchao Dou, Zhendong Sun, Depeng Shen, Ning Guo, Zhe Liu, Lin Cheng, Yongchao Liu, Bingtao Tang

**Affiliations:** 1 School of Mechanical Engineering, Qilu University of Technology (Shandong Academy of Sciences), Jinan 250353, China; 2 Shandong Machinery Design & Research Institute, Jinan 250031, China; 3 Jinlei Technology Co., Ltd., Jinan 271104, China

**Keywords:** Fe-Cr-Mo-Mn steel, hot deformation, constitutive model, hot processing map, microstructural evolution

## Abstract

The Laasraoui segmented and Arrhenius flow stress model, dynamic recrystallization (DRX) model, grain size prediction model, and hot processing map (HPM) of Fe-Cr-Mo-Mn steels were established through isothermal compression tests. The models and HPM were proven by experiment to be highly accurate. As the deformation temperature decreased or the strain rate increased, the flow stress increased and the grain size of the Fe-Cr-Mo-Mn steel decreased, while the volume fraction of DRX (*X*_drx_) decreased. The optimal range of the hot processing was determined to be 1050–1200 °C/0.369–1 s^−1^. Zigzag-like grain boundaries (GBs) and intergranular cracks were found in the unstable region, in which the disordered martensitic structure was observed. The orderly packet martensite was formed in the general processing region, and the mixed structure with incomplete DRX grains was composed of coarse and fine grains. The microstructure in the optimum processing region was composed of DRX grains and the multistage martensite. The validity of the Laasraoui segmented flow stress model, DRX model, grain size prediction model, and HPM was verified by upsetting tests.

## 1. Introduction

Fe-Cr-Mo-Mn steels exhibit exceptional strength, excellent hardenability, superior fatigue resistance, and remarkable impact toughness at low temperatures, and they are capable of withstanding alternating loads [1,2,3,4], making them extensively utilized in wind power, nuclear energy, and other industries [5,6,7]. Hot forging is the most widely used method for the fabrication of steel-based components, and it has characteristics of a high production efficiency and good product quality [8,9]. However, billets are susceptible to cracking and other issues during this process. To avoid these shortcomings, the influence of hot-forming parameters on the hot deformation behavior and mechanism should be systematically studied [10].

The behavior and mechanism of hot deformation are typically predicted using the constitutive model and HPM [11,12], which were established based on the dynamic material model (DMM), Laasraoui segmented model, and DRX model. The parameters needed to build these models, such as instability coefficient, power dissipation coefficient, stress (*σ*), steady state stress ($\sigma_{ss}$), yield stress ($\sigma_{0}$), turning strain ($\varepsilon_{c}$), and peak strain ($\;\varepsilon_{p}$), are determined based on compression tests [13,14,15]. The HPM is typically categorized into instability regions, general processing regions, and optimal processing regions. Forging defects such as cracks, adiabatic shear bands, and localized flow usually occur in the instability region [16]. The dynamic recovery (DRV) and DRX occur in the general processing region, while complete DRX occurs in the optimal processing region.

Currently, numerous scholars have conducted extensive research on the hot deformation characteristics of steel based on HPM and the constitutive model. Zhu [17] modified the constitutive model based on the Zerilli–Armstrong model to describe the dynamic mechanical behavior of 42CrMo steel. Li [18] constructed a hyperbolic sine-type equation based on the Zener–Hollomon (*Z*) parameter. Quan [19] predicted the high-temperature deformation behavior of 42CrMo by utilizing the back propagation learning algorithm of an artificial neural network. Lin [20] constructed the flow stress constitutive equations by using the hyperbolic sine function. Kim [21] developed a constitutive and DRX model based on hot torsion and compression tests. Qi [22] established the processing diagram and optimized the forging parameters. Ji [23] constructed the functional relationship between the relevant material constants and the *Z* parameter under 0.2–0.8 strains. These research methods and approaches have also been widely employed in other steels. Liu [24] explored the hot deformation behavior of FGH4096 and established a new grain size model. Zhou [25] constructed HPM of BG801-bearing steel and determined the optimal hot working process. The mechanism of hot deformation can be further revealed through the characterization of the microstructure. Deformations at high temperatures usually produce a large number of substructures, which are retained after rapid cooling [26]. A direct observation and analysis of the evolution of austenite substructures after hot deformation is difficult due to the phase transformation in the cooling process [27]. For this reason, special austenitic steel was selected to reveal the substructure after hot deformation, which can completely retain the high-temperature structure after cooling [28,29,30]. The results indicated that high-density dislocations in deformed austenitic promote the nucleation of acicular ferrite, while the lamellar bainite and martensite tend to form in DRX austenitic. Therefore, hot deformation changes the kinetics of phase transition and the structure of martensite. Compared with austenitic steel, the mechanical properties of tempered steel are significantly influenced by the martensitic structure [31]. Prawoto [32] studied how austenitizing affects the microstructure and morphology of tempered steel, revealing that the dislocation caused by the transformation from austenite to martensite could significantly influence its mechanical properties. Wang [33] investigated the influence of a hot-deformed substructure on phase transition in tempered steel and observed that a portion of this substructure was inherited in deformed austenite.

Although many scholars have explored the hot deformation behavior of steel, these studies primarily focus on the constitutive model of the rheological behavior and the DRX model. Moreover, they also ignore the microstructural characteristics of different processing regions in the HPMs, which are usually combined with the high-temperature microstructural characteristics of austenite. The effect of martensitic transformation microstructure at room temperature is ignored. In addition, the microstructural activity relationship between instability rate, power dissipation rate, and room temperature microstructure after martensitic transformation has been rarely reported. Furthermore, most researchers have only obtained HPMs without verification.

In this study, an Fe-Cr-Mo-Mn steel for large forgings of a wind turbine spindle was taken as the research material. As a kind of low-alloy hypoeutectoid steel, it has a high strength and good hardenability, and it can withstand the impact of high alternating loads. Combined with the hot deformability characteristics of the Fe-Cr-Mo-Mn steel, the influence of temperature and strain rate on the flow stress and microstructure of the Fe-Cr-Mo-Mn steel was studied, and the influence of hot working technology on the Fe-Cr-Mo-Mn steel-forming mechanism was revealed. This is of great significance for optimizing hot working technology and improving the forming quality of Fe-Cr-Mo-Mn steels. In this study, it was found that the parameters of the hot deformation, like instability rate and power dissipation, are closely associated with DRX and martensitic transformation. The high-temperature flow stress model, DRX model, and HPMs under different strains were constructed, and optimal hot working parameters for Fe-Cr-Mo-Mn steels were identified. The microstructural evolution of the Fe-Cr-Mo-Mn steel under different processing regions in the HPMs was studied based on DRX and martensite transformation. In addition, the accuracy of the proposed models and established HPMs was verified by upsetting tests at a high temperature and numerical simulations. This work could provide valuable insights to draw up hot working technology routes for the industrialized production of Fe-Cr-Mo-Mn steels.

## 2. Materials and Methods

**Figure 1:**
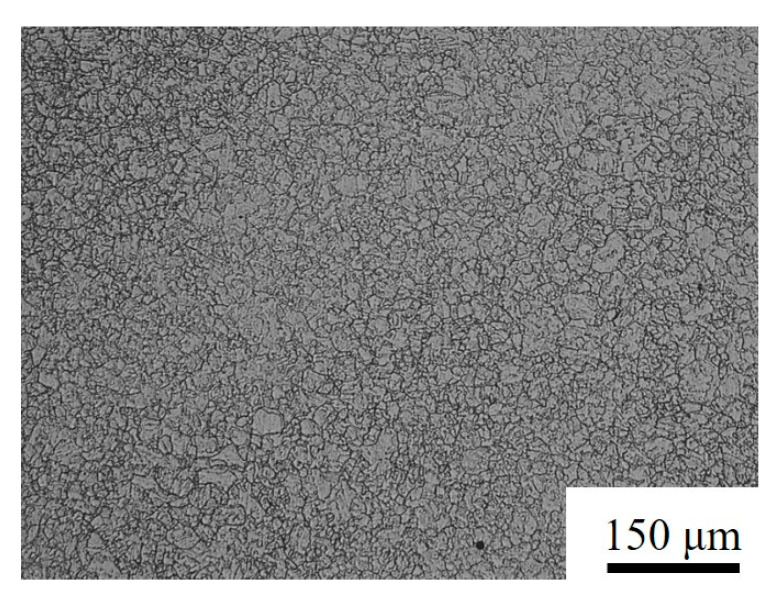
The microstructure of the austenitized Fe-Cr-Mo-Mn steel.

Cylindrical specimens of an Fe-Cr-Mo-Mn steel with a diameter of 10 mm and a height of 14 mm were used for high-temperature compression tests. This steel was achieved by adjusting the alloy composition as well as the melting and casting processes to achieve the targeted chemical compositions. The chemical composition of the steel is presented in Table 1. Figure 1 shows the microstructure of austenitized Fe-Cr-Mo-Mn steel exhibiting an equiaxial austenitic structure; the average grain size is 22.87 μm.

**Figure 2:**
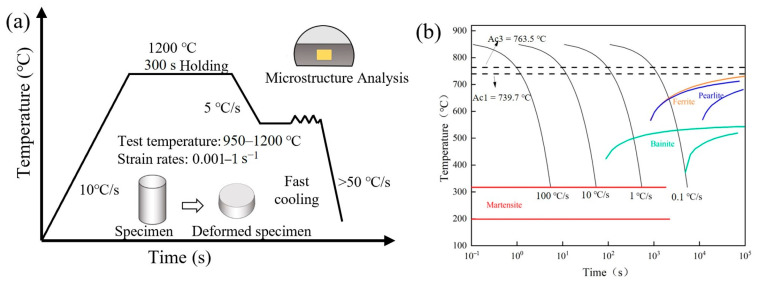
(**a**) Schematic of compression test; (**b**) CCT curve of Fe-Cr-Mo-Mn steel.

The high-temperature compression experiment was conducted using the Gleeble thermomechanical simulator. Initially, the specimen was heated to 1200 °C at a rate of 10 °C/s and preserved for 300 s. Subsequently, the deformation temperatures were gradually decreased to 950 °C, 1000 °C, 1050 °C, 1100 °C, and 1200 °C, respectively, with a cooling rate of 10 °C/s, and preserved for 30 s. Then, compression experiments were conducted with different strains (60% and 40%) and strain rates (0.001 s^−1^, 0.01 s^−1^, 0.1 s^−1^, 1 s^−1^). The specimen was finally rapidly cooled to room temperature at a rate of >50 °C/s to ensure that the microstructure of the sample was completely transformed from austenite to martensite. The continuous cooling transformation (CCT) curve of Fe-Cr-Mo-Mn steel is shown in Figure 2b. The deformed specimen was cut linearly along the axis and etched using a mixture of supersaturated picric acid, sodium dodecyl benzene sulfonate (water bath with 60–70 °C), and 4% nitric acid–alcohol solution. The EBSD specimens were obtained through electrolytic polishing using a 10% perchloric acid ethanol solution as the electrolyte. A Zeiss Axio Imager 2 OM optical microscope (OM) (Zeiss, Oberkochen, Germany), a SU3500 scanning electron microscope (SEM) (Hitachi, Tokyo, Japan), and a Zeiss 300 electron back scatter diffraction (EBSD) (Zeiss, Oberkochen, Germany) were used to analyze the microstructure. The EBSD data were analyzed using Channel 5 software. The average grain size was measured and calculated by employing the intercept method. The experimental process route and microstructural characterization position are depicted in Figure 2a.

## 3. Results and Discussion

### 3.1. Flow Characteristics and Deformation Mechanisms of Fe-Cr-Mo-Mn Steel

**Figure 3:**
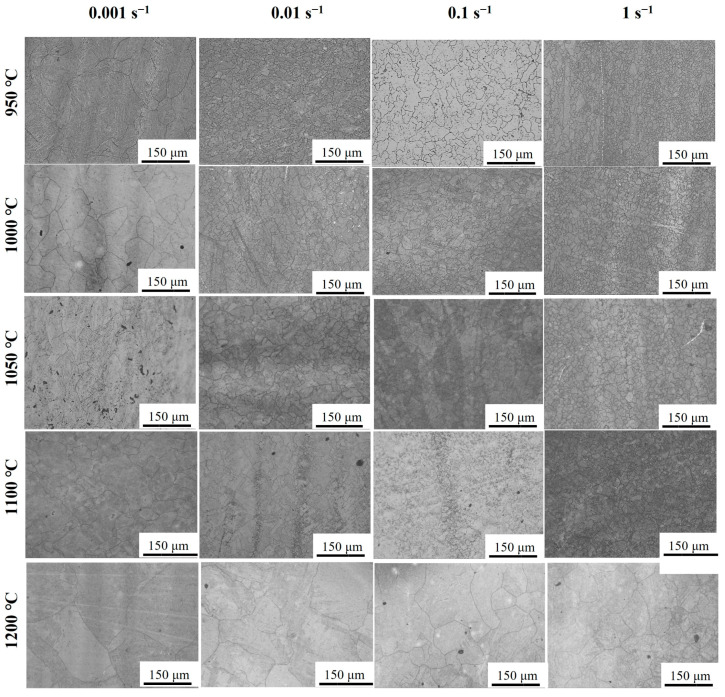
OM of deformed Fe-Cr-Mo-Mn steel at a strain of 0.92 under different deformation conditions.

Figure 3 shows the microstructure of an Fe-Cr-Mo-Mn steel under different deformation temperatures and strain rates. The grain size decreased as the strain rate increased or the deformation temperature decreased. Notably, the average grain size exhibited a significant increase as the temperature rose from 1100 °C to 1200 °C. The rapid increase in size is attributed to the gradual dissolution of carbides within the microstructures, which results from elevated temperatures and the elimination of carbide-pinning effects at GBs [23,25].

**Figure 4:**
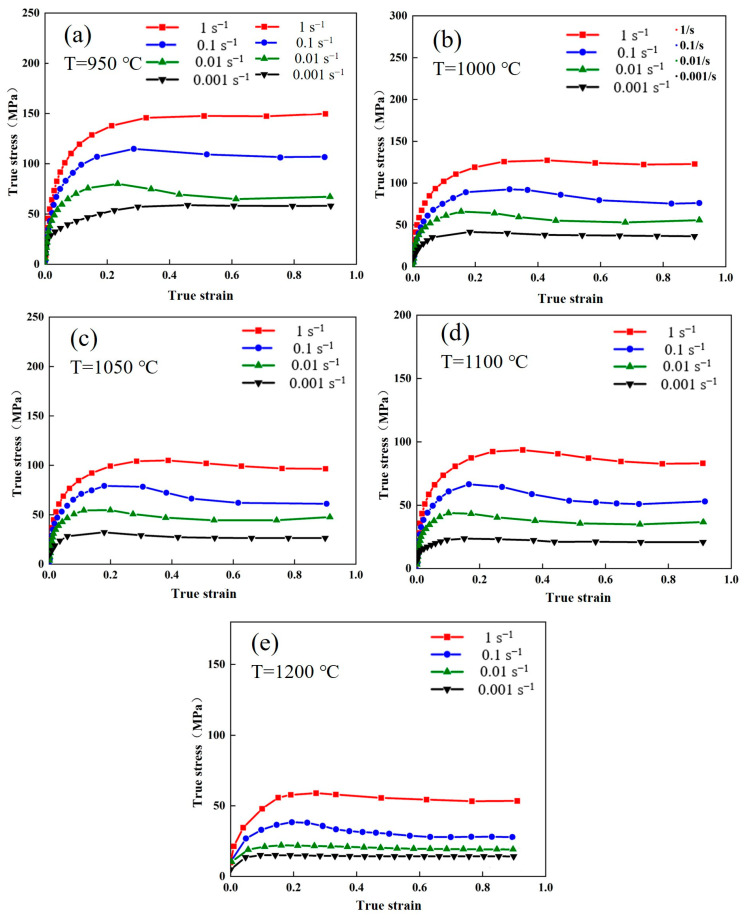
The flow stress–strain curves of the Fe-Cr-Mo-Mn steel: (**a**) 950 °C, (**b**) 1000 °C, (**c**) 1050 °C, (**d**) 1100 °C, (**e**) 1200 °C.

The flow stress–strain curve of the Fe-Cr-Mo-Mn steel under various hot deformation conditions is shown in Figure 4. The flow stress–strain curves exhibit two different characteristics under varying hot deformation conditions. In the first type of curve, the stress gradually increases with the increase in strain and tends to stabilize after reaching its peak value. The characteristic curve mentioned can be observed under the temperature range of 950 °C and the strain rate range of 0.01 s^−1^ (950 °C/0.01 s^−1^). In the second type, the flow stress exhibits a rapid increase with increasing strain, followed by a gradually decrease after reaching its peak value, and it finally stabilizes. The curves of the hot deformation conditions of 1050 °C/0.001–1 s^−1^ could exemplify this type. During the initial deformation stage, the increase in flow stress with the increase in strain is attributed to dislocation multiplication occurring within the material [34,35,36]. The flow stress gradually increases as the strain further increases, which can be attributed to dislocation annihilation resulting from both the slip and climb of dislocations. When the peak strain is reached, recrystallization softening occurs. The above two characteristic flow stress–strain curves are eventually generated through the combined effects of softening and work hardening (WH).

Based on Figure 4, the WH effect is enhanced at the same deformation temperature, while the peak stress increases with the increase in strain rate. Conversely, under the same deformation rate, the peak stress decreases with the increase in the deformation temperature. It also shows that DRX can easily occur when the temperature increases. The acceleration of vacancy in the atoms diffused at higher temperatures enhances the dislocation slip and climb, thereby promoting DRX. However, a higher strain rate results in the reduction of material energy storage and insufficient time for nucleation and grain growth, inhibiting DRX occurrence. Luo et al. [37] demonstrated that DRX grain nucleation and growth are facilitated at high temperatures compared to low deformation temperatures. Liu et al. [38] showed that low strain rates extend the deformation period for DRX evolution, which can completely resist the WH effect. It can be concluded that with the increase in deformation temperature and decrease in strain rate, DRX is promoted, and the flow stress of the material is reduced.

In the process of hot compression, with the occurrence of deformation, the rise in temperature and friction may affect the results, but whether the error is caused by the rise in temperature and friction needs to be determined.

**Figure 5:**
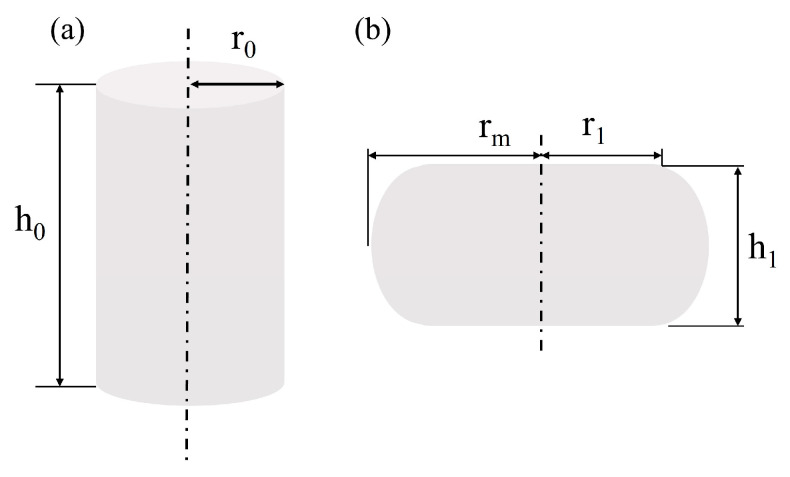
Schematic diagram of the shape of the sample before and after compression deformation at a high temperature: (**a**) before compression deformation; (**b**) after compression deformation.

The expansion coefficient *B*_p_ is usually used to determine if friction correction is needed for a stress–strain curve:

(1) $$B_{p}=\frac{h_{1}r_{m}^{2}}{h_{0}r_{0}^{2}}$$

where, ℎ_0_ is the height of the specimen before compression; ℎ_1_ is the height of the specimen after compression; *r*_0_ is the radius before compression; *r*_1_ is the radius of the end face after compression; and *r*_m_ is the maximum radius after compression. The shape of the specimen before and after compression deformation at high temperatures is shown in Figure 5.

When *B*_p_ ≤ 1.1, it indicates that the experiment is unaffected by friction and the obtained stress–strain is considered acceptable. When *B*_p_ > 1.1, it suggests that friction significantly influences the experiment and friction correction is required. The *B*_p_ of the Fe-Cr-Mo-Mn steel at different hot deformation conditions was calculated using Formula (1), and the results are shown in Table 2.

The calculated results show that *B*_p_ is ≤ 1.1. Therefore, friction did not need to be corrected.

Temperature correction is obtained using Formula (2):

(2) $$\sigma_{t}\approx \frac{T}{T_{t}}\sigma$$

where *σ*_t_ is the actual stress after temperature rise correction, *T* is the experimental temperature, *T_t_* is the set deformation temperature, and *σ* is the experimental stress.

At the same strain rate, the flow stress gradually decreases with the increase in the deformation temperature; the temperature rise of the sample at the same deformation rate is slightly affected by the actual temperature. As can be seen from Formula (2), the greater the set deformation temperature *T_t_*, the lower the influence of temperature rise on the actual stress value. And due to the lower stress value at high temperatures, the stress value is lower. Therefore, the effect of temperature rise on the deformation of Fe-Cr-Mo-Mn steels under high temperature conditions can be ignored.

### 3.2. Construction of the Arrhenius Model

The constitutive equation model is based on the Arrhenius model [39], and it is used to describe the relationship between hot deformation activation energy, deformation temperature, and strain rate.

To study the impact of temperature and strain rate on the rheological behavior, the *Z* parameter was introduced, as shown in Formula (3) [40]:

(3) $$Z=\dot{\varepsilon }\exp\left(\frac{Q}{RT}\right)$$

where *Z* denotes the strain rate factor of temperature compensation, *Q* denotes hot deformation activation energy, $\dot{\varepsilon }$ is strain rate, and *R* (8.314 J/(mol·K)) denotes a gas constant.

According to the hyperbolic sine function relationship proposed by Sellars [41], there are three different formulas (Formulas (4)–(6)) in the hyperbolic sine function according to stress. The relationship between different σ, *T*, and $\dot{\varepsilon }$ can be obtained.

(4) $$\dot{\varepsilon }=A_{1}\sigma^{n_{1}}\exp\left(-\frac{Q}{RT}\right)\;\alpha \sigma <0.8$$

(5) $$\dot{\varepsilon }=A_{2}\exp(\beta \sigma )\left(-\frac{Q}{RT}\right)\;\alpha \sigma <1.2$$

(6) $$\;\dot{\varepsilon }=A[{sinh(\alpha \sigma )]}^{n_{2}}\exp\left(-\frac{Q}{RT}\right)\;All\;stress$$

where *A*_1_, *A*_2_, *A*, *n*_1_, *n*_2,_ and *β* are material constants; *α* = *β/n*_1_.

Formulas (4)–(6) can be reformulated in terms of the *Z* parameter through logarithmic transformation, as represented by Formulas (7)–(9).

(7) $$\;lnZ=ln\dot{\varepsilon }+\frac{Q}{RT}=lnA_{1}+n_{1}ln\sigma \;\;\;\alpha \sigma <0.8$$

(8) $$lnZ=ln\dot{\varepsilon }+\frac{Q}{RT}=lnA_{2}+\beta \sigma \;\alpha \sigma <1.2$$

(9) $$lnZ=ln\dot{\varepsilon }+\frac{Q}{RT}=lnA+n_{2}lnsinh(\alpha \sigma )\;All\;stress$$

Formulas (3) and (6) are combined to calculate the partial derivative of ln$\dot{\varepsilon }$ and 1/*T*, obtaining Formulas (10) and (11):

(10) $$\;n={\left[\frac{\partial \ln\dot{\varepsilon }}{\partial \ln\left(\sinh\left(\alpha \sigma \right)\right)}\right]}_{T=const}$$

(11) $$Q={Rn\left[\frac{\partial \ln\left(\sinh\left(\alpha \sigma \right)\right)}{\partial \left(1/T\right)}\right]}_{\overset{.}{\varepsilon }=const}$$

McQueen [42] pointed out that $\sigma_{p}$ or σ_ss_ can be used to establish the high-temperature flow stress model, including the dynamic recrystallization stage. Because $\sigma_{p}$ is easier to obtain than σss, the usual choice is to use $\sigma_{p}$ to calculate *Q*.

**Figure 6:**
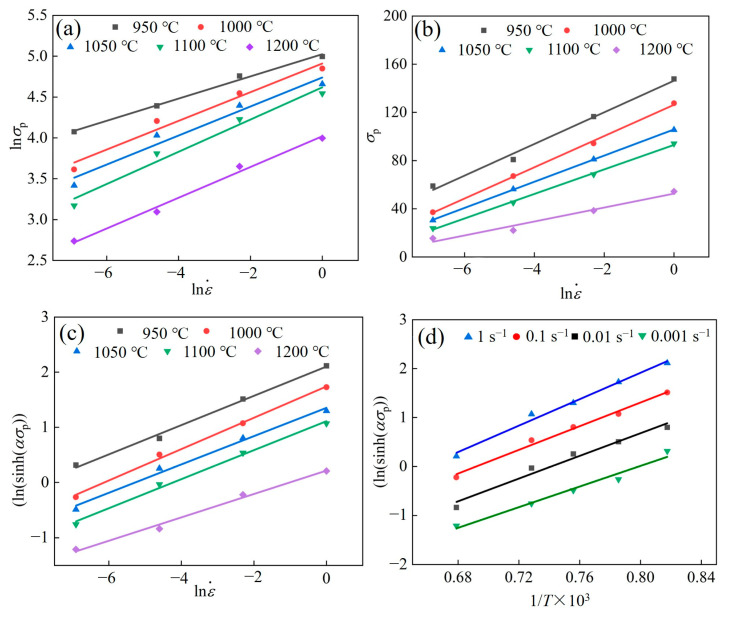
(**a**) ln$\sigma_{p}-\ln\dot{\varepsilon }$, (**b**) $\sigma_{p}-\ln\dot{\varepsilon }$, (**c**) $lnsinh(\alpha \sigma_{p})-\ln\dot{\varepsilon }$, (**d**) $lnsinh(\alpha \sigma_{p})-$1/*T*×1000.

According to the slope of the fitted line in Figure 6, the values of *n*_1_, *β*, and *α* were calculated to be 5.4288, 0.103316, and 0.10903, respectively. The average slope of $\ln\left(\sinh\left(\alpha \sigma \right)\right)$−1/T was calculated to be 3.9446; the average slope of $\ln\left(\sinh\left(\alpha \sigma \right)\right)-1/T$ was calculated to be 11.9217.

Formula (12) can be obtained by combining Formulas (10) and (11):

(12) $$Q=R{\left[\frac{\partial \ln\dot{\varepsilon }}{\partial \ln\left(\sinh\left(\alpha \sigma_{p}\right)\right)}\right]}_{T=constant}{\left[\frac{\partial \ln\left(\sinh\left(\alpha \sigma_{p}\right)\right)}{\partial (1/Τ)}\right]}_{\overset{.}{\varepsilon }=constant}$$

**Figure 7:**
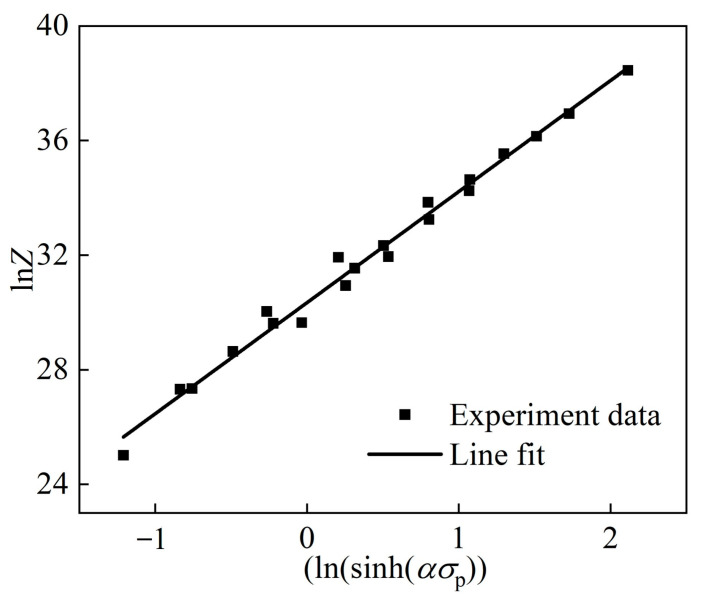
Relationship between ln*Z* and ln(sinh(*ασ*_p_)).

In summary, the *Q* of the Fe-Cr-Mo-Mn steel was calculated as 382.5122 kJ/mol. The *Z* parameter can be obtained by inserting the returned *Q* into Formula (3) and plotting Figure 7. By further transforming the *n*_2_ and *A* values using mathematical methods, more accurate values can be obtained: *n*_2_ = 3.8772, *A* = 1.5042 × 10^13^. Combining Formula (3) with Formula (6), the relationship between the $\sigma_{p}$, *T*, and $\dot{\varepsilon }$ can be obtained, as shown in Formula (13):

(13) $$\dot{\varepsilon }=1.5042\times 10^{13}{\left(\sinh\left(0.10903\sigma_{p}\right)\right)}^{3.8772}\exp\left(-\frac{382,512.2}{8.314T}\right)$$

In order to predict the hot behavior of the Fe-Cr-Mo-Mn steel more accurately, the effect of strain on the constitutive relationship was taken into account. Arrhenius used a fifth-order polynomial for fitting, as shown in Formula (14):

(14) $$\;\left\{\begin{array}{c} \alpha =X_{0}+X_{1}\;\varepsilon +X_{2}\;\varepsilon^{2}+X_{3}\;\varepsilon^{3}+X_{4}\;\varepsilon^{4}+X_{5}\;\varepsilon^{5}\\ n=N_{0}+N_{1}\varepsilon +N_{2}\varepsilon^{2}+N_{3}\varepsilon^{3}+N_{4}\varepsilon^{4}+N_{5}\varepsilon^{5}\\ Q=Q_{0}+Q_{1}\varepsilon +Q_{2}\varepsilon^{2}+Q_{3}\varepsilon^{3}+Q_{4}\varepsilon^{4}+Q_{5}\varepsilon^{5}\\ lnA=Y_{0}+Y_{1}\varepsilon +Y_{2}\varepsilon^{2}+Y_{3}\varepsilon^{3}+Y_{4}\varepsilon^{4}+Y_{5}\varepsilon^{5} \end{array}\right\}$$

**Figure 8:**
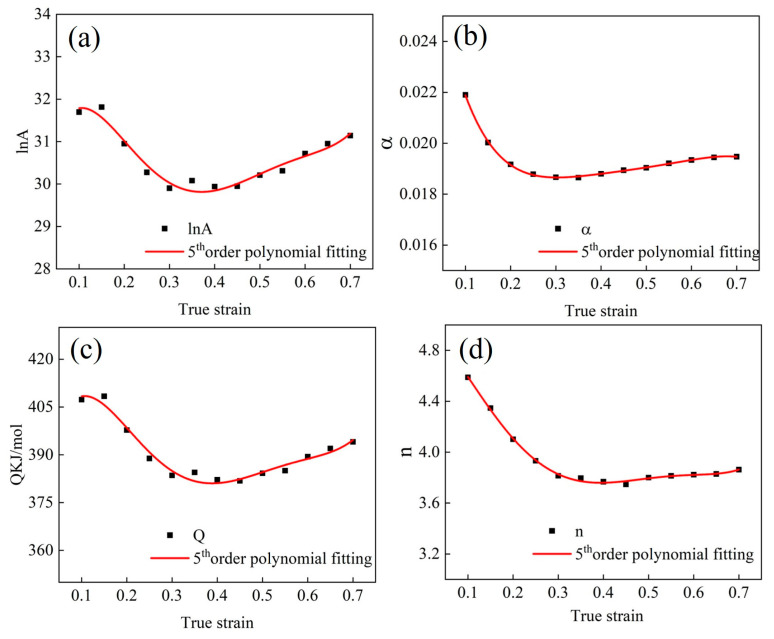
Fifth-order polynomial fitting of the relationship between material constant ((**a**) ln*A*; (**b**) *α*; (**c**) *Q*; (**d**) *n*) and true strain.

The fifth-order polynomial regression was performed on the data in Table 3, and the fitting curves are shown in Figure 8. The fitted polynomial parameters are shown in Table 4.

**Figure 9:**
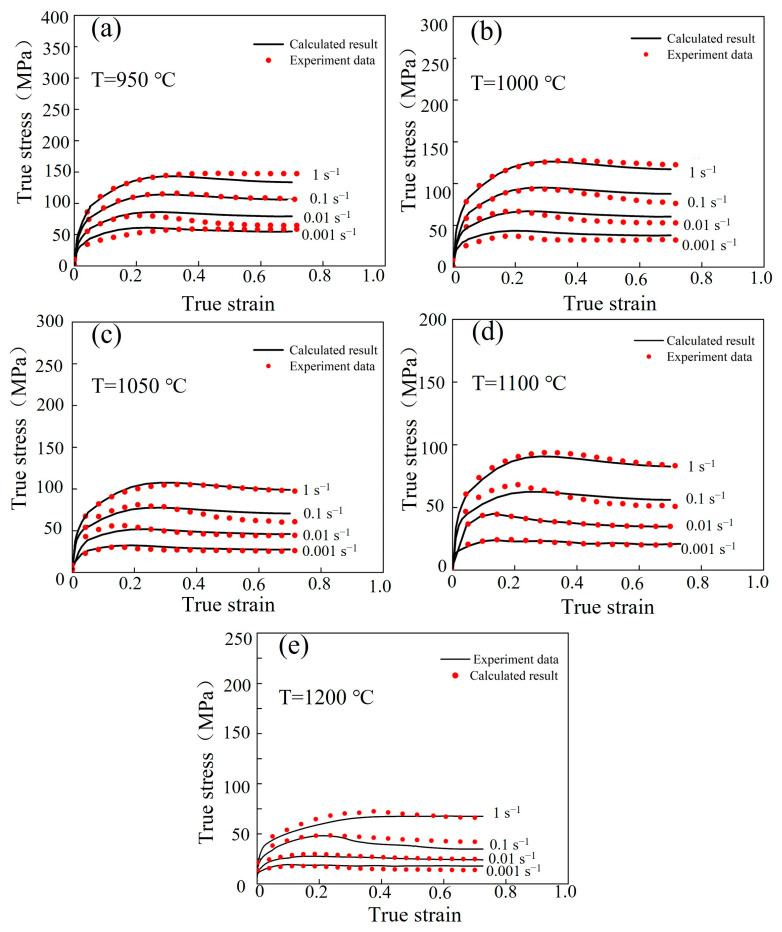
Comparing the calculated and experimental results of the Arrhenius model for the Fe-Cr-Mo-Mn steel: (**a**) 950 °C, (**b**) 1000 °C, (**c**) 1050 °C, (**d**) 1100 °C, (**e**) 1200 °C.

Formulas (3), (6) and (13) were combined to obtain Formula (15), the flow stress values under different strains were calculated, and the calculated values were compared with the experimental values, as shown in Figure 9. The Arrhenius model was obtained with a high accuracy.

(15) $$\sigma =\frac{1}{\alpha (\varepsilon )}ln\left\{{\left(\frac{\overset{.}{\varepsilon }\exp\left(\frac{Q(\varepsilon )}{RT}\right)}{A(\varepsilon )}\right)}^{\frac{1}{n(\varepsilon )}}+{\left[{\left(\frac{\overset{.}{\varepsilon }\exp\left(\frac{Q(\varepsilon )}{RT}\right)}{A(\varepsilon )}\right)}^{\frac{1}{n(\varepsilon )}}+1\right]}^{\frac{1}{2}}\right\}$$

### 3.3. Construction of the Laasraoui Segmented and DRX Models

DRX usually occurs during the hot forming process, which significantly affects both the microstructure and the yield stress characteristics. The Arrhenius model cannot accurately describe the changes in internal physical variables such as DRX during material deformation. This is based on the calculation basis for the Arrhenius model in Section 3.2. The Laasraoui segmented model with DRX was used in this study to model the high-temperature flow stress–strain curve of the Fe-Cr-Mo-Mn steel, as shown in Formula (16):

(16) $$\left\{\begin{array}{l} \begin{array}{l} \sigma_{WH}={\left[\sigma_{s}^{2}+\left(\sigma_{0}^{2}-\sigma_{s}^{2})e^{-\Omega \varepsilon }]\right.\right.}^{0.5},\;\;\;\varepsilon <\varepsilon_{c}\\ \sigma =\sigma_{WH}-\left(\sigma_{s}-\sigma_{ss}\right)\left\{1-exp\left[-k_{d}{\left(\frac{\varepsilon -\varepsilon_{c}}{\varepsilon_{p}}\right)}^{n_{d}}\right]\right\},\;\;\;\varepsilon \geq \varepsilon_{c} \end{array} \end{array}\right.$$

where $\sigma_{WH}$ is the stress at the stage of WH, Ω is the dynamic softening coefficient, $k_{d}$, $n_{d}$ are the material constants.

**Figure 10:**
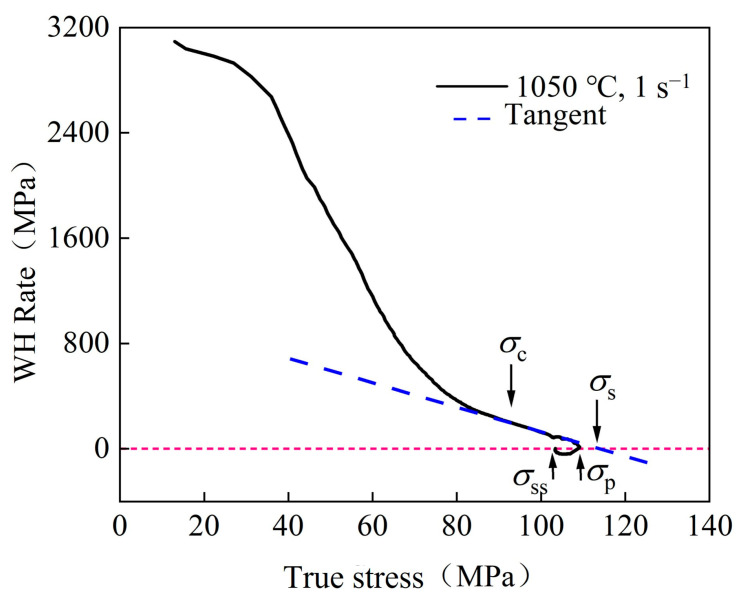
WH rate curve.

**Figure 11:**
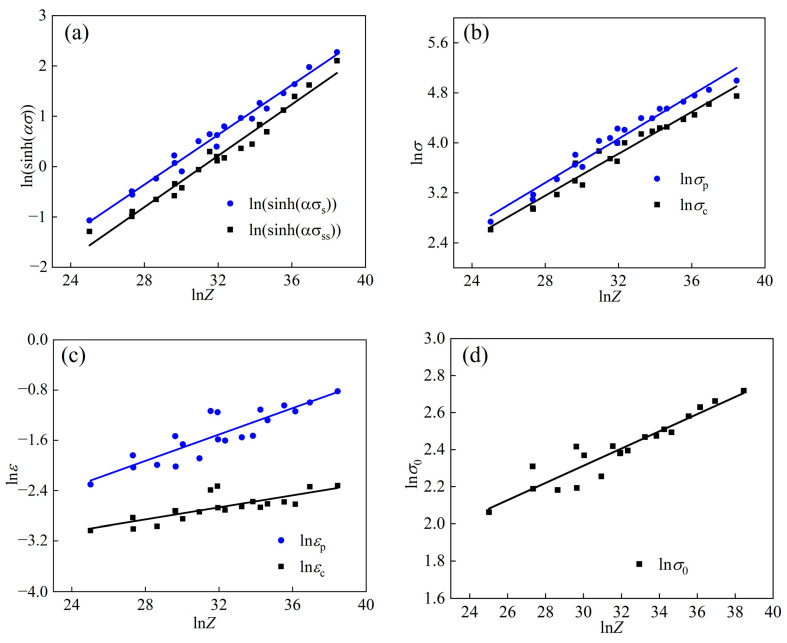
The relationships between material parameters and the *Z* parameter (a) lnsinh(ασ)–ln⁡Z, (b) lnσ–ln⁡Z, (c) lnε–ln⁡Z, (d) $ln\sigma_{0}–\ln Z$.

Figure 10 shows the $\theta \left(\theta =\partial \sigma /\partial \varepsilon \right)-\sigma$ curve of the WH rate under the 1050 °C/1 s^−1^ condition. The material’s $\sigma_{c}$, $\sigma_{p}$, $\sigma_{s}$, and $\sigma_{ss}$ were acquired based on the WH rate curve under various temperatures and strain rates. Table 5 shows the specific values of parameters required for the Laasraoui segmented flow stress model at different temperatures and strain rates, which are obtained from the WH rate curves and stress–strain curves. The correlation between $\sigma_{c}$ and $\varepsilon_{c}$ on *Z* can be obtained from the linear regression in Figure 11b,c. The results indicate that both $\sigma_{c}$ and $\varepsilon_{c}$ exhibit an upward trend as the Z value increases, corresponding to the decrease in temperature or the increase in the strain rate. This property can, thus, be effectively represented by the *Z* parameter function. Similarly, other parameters also exhibited analogous relationships. The correlation between these parameters and the *Z* parameter is shown in Figure 11, which can be mathematically expressed by Formulas (17)–(23).

(17) $$\sigma_{s}=52.5458\times \sinh^{-1}\left(6.8312\times 10^{-4}Z^{0.24753}\right)$$

(18) $$\sigma_{ss}=52.5458\times \sinh^{-1}\left(3.5433\times 10^{-4}Z^{0.25502}\right)$$

(19) $$\varepsilon_{p}=0.007692Z^{0.1052}$$

(20) $$\varepsilon_{c}=0.014997Z^{0.04787}$$

(21) $$\sigma_{p}=0.2195Z^{0.1752}$$

(22) $$\sigma_{c}=0.2159Z^{0.1674}\;$$

(23) $$\sigma_{0}=2.507033Z^{0.04646}$$

The critical strain of DRX during hot deformation can be derived from stress–strain curves [43,44].

The flow stress curve can be used to estimate *X*_drx_ as the experimental value, as shown in Formula (24):

(24) $$X_{drx}=\frac{\sigma_{WH}-\sigma }{\sigma_{s}-\sigma_{ss}},\;\;\;(\varepsilon >\varepsilon_{c})$$

Ω can be determined by Formula (25):

(25) $$\sigma_{WH}={\left[\sigma_{s}^{2}+\left(\sigma_{0}^{2}-\sigma_{s}^{2}\right)e^{-\Omega \varepsilon }\right]}^{0.5},\;\;\;\varepsilon <\varepsilon_{c}$$

The point of WH is selected from the flow stress curves, and its stress and strain values are substituted into Formula (26) to construct the lnΩ–lnZ relationship diagram. Following fitting, the mathematical expression of Ω is determined as follows:

(26) $$\Omega =55.2175Z^{-0.05469}$$

The DRX model is typically represented by calculated values that are encoded in the DEFORM v11 software’s pre-processing, as shown in Formula (27) [45]:

(27) $$X_{drx}=1-\exp\left[-k_{d}{\left(\frac{\varepsilon -\varepsilon_{c}}{\varepsilon_{p}}\right)}^{n_{d}}\right]$$

**Figure 12:**
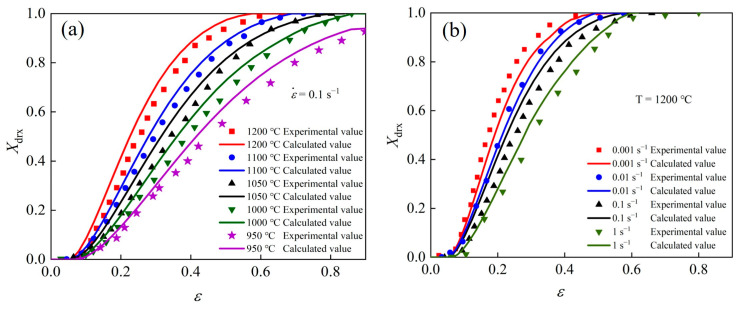
The comparison between the *X*_drx_ calculated by models and those obtained through experiments: (**a**) strain rate = 0.1 s^−1^, (**b**) temperature = 1200 °C.

The established DRX model is consistent with the experimental results, as shown in Figure 12.

**Figure 13:**
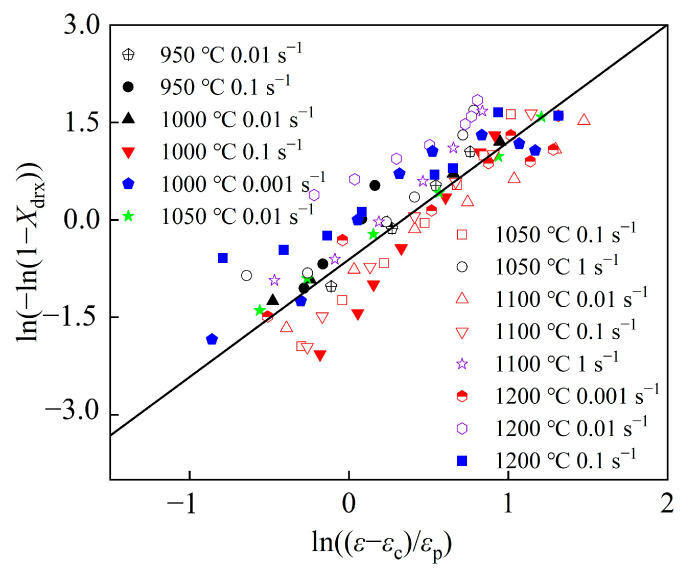
Schematic diagram of the DRX percentage model of the Fe-Cr-Mo-Mn steel.

In Figure 13, the values of $k_{d}$ and $n_{d}$ are respectively fitted to be 0.673054 and 1.61985, thus obtaining the DRX model of the material.

In summary, considering the strain rate, using 0.1 s^−1^ as an example, the higher the temperature, the earlier the DRX. At the same temperature, using 1200 °C as an example, the lower the strain rate, the earlier the DRX. Therefore, increasing the temperature or decreasing the strain rate is beneficial to DRX.

The Laasraoui segmented model and DRX model of the Fe-Cr-Mo-Mn steel are expressed by Formula (28):

(28) $$\left\{\begin{array}{c} \sigma_{WH}={\left[\sigma_{s}^{2}+\left(\sigma_{0}^{2}-\sigma_{s}^{2})e^{-\Omega \varepsilon }]\right.\right.}^{0.5},\;\;\;\varepsilon <\varepsilon_{c}\\ \sigma =\sigma_{WH}-\left(\sigma_{s}-\sigma_{ss}\right)\left\{1-exp\left[-k_{d}{\left(\frac{\varepsilon -\varepsilon_{c}}{\varepsilon_{p}}\right)}^{n_{d}}\right]\right\},\;\;\;\varepsilon \geq \varepsilon_{c}\\ X_{drx}=1-\exp\left[-0.673054{\left(\frac{\varepsilon -\varepsilon_{c}}{\varepsilon_{p}}\right)}^{1.61985}\right]\;\\ Z=\dot{\varepsilon }exp(\frac{382,512.232}{\mathrm{RT}})\\ \sigma_{s}=52.5458\times \sinh^{-1}\left(6.8312\times 10^{-4}Z^{0.24753}\right)\\ \sigma_{ss}=52.5458\times \sinh^{-1}\left(3.5433\times 10^{-4}Z^{0.25502}\right)\\ \varepsilon_{p}=0.007692Z^{0.1052}\\ \;\;\varepsilon_{c}=0.014997Z^{0.04787}\\ \Omega =21.1521Z^{-0.02149} \end{array}\right.$$

**Figure 14:**
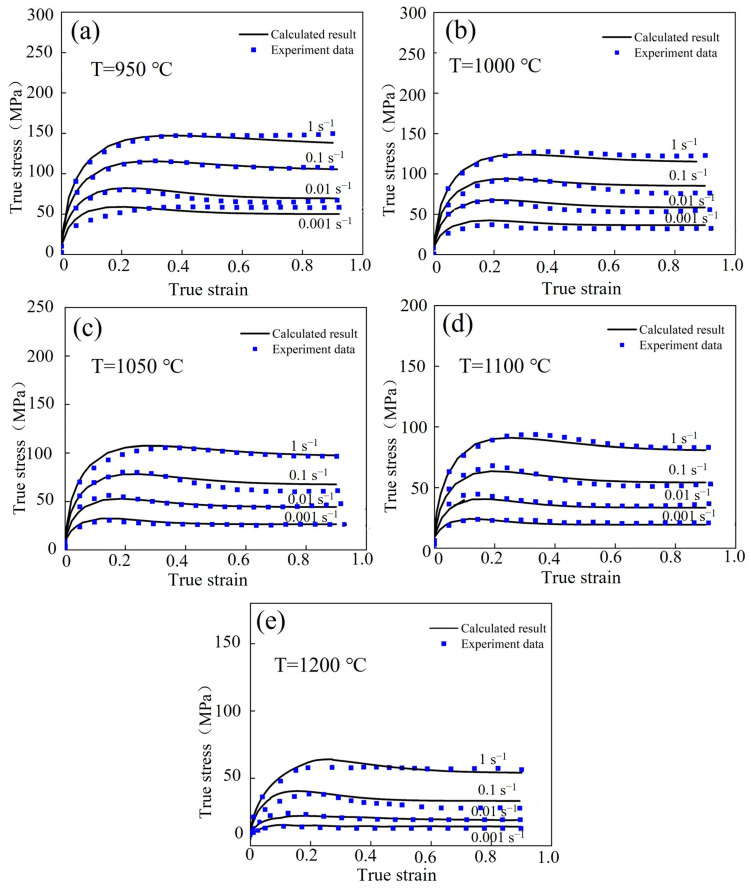
Comparing the calculated and experimental results of Laasraoui segmented model for the Fe-Cr-Mo-Mn steel: (**a**) 950 °C, (**b**) 1000 °C, (**c**) 1050 °C, (**d**) 1100 °C, (**e**) 1200 °C.

The flow stress values were estimated using the Laasraoui segmented model under various deformation situations. The results of the numerical simulation were highly in accordance with those of the experiments, as shown in Figure 14.

The Arrhenius and Laasraoui segmented models’ accuracy was assessed by calculating the relative coefficient (*R*) and the average absolute relative error (AARE) [11]. Among these, the smaller the AARE value, the higher the accuracy of the models’ prediction. The calculation is shown in Formulas (29) and (30):

(29) $$R=\frac{\sum_{i=1}^{N}{\left(E_{i}-\bar{E}\right)}^{\;}\left(P_{i}-\bar{P}\right)}{\sqrt{\sum_{i=1}^{N}{\left(E_{i}-\bar{E}\right)}^{2}\sum_{i=1}^{N}{\left(P_{i}-\bar{P}\right)}^{2}}}$$

(30) $$AARE\left(\%\right)=\frac{1}{N}\sum_{i=1}^{N}\left|\frac{E_{i}-P_{i}}{E_{i}}\right|$$

where *N* is the total stress value used in the experiment, $E_{i}$ is the experimental stress value, $\bar{E}$ is the average value of the experimental stress, $P_{i}$ is the predicted value of the stress, and $\bar{P}$ is the average value of the predicted stress of the model.

**Figure 15:**
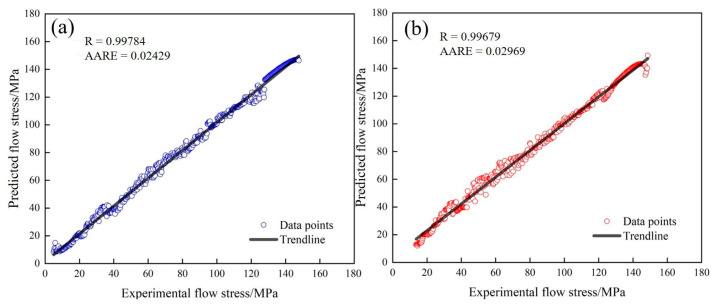
Comparison of experimental values with predicted values: (**a**) Laasraoui model; (**b**) Arrhenius model.

The correlation between the stress predicted and the experimental values obtained by the Laasraoui segmented and Arrhenius model were compared and analyzed, as shown in Figure 15. The value of *R* was as high as 0.99784 and 0.99679, and the value of AARE was as low as 0.0242 and 0.02969, indicating the high accuracy of the two models. Although the Arrhenius model can accurately predict the relationship between strain, strain rate, temperature, and flow stress of the Fe-Cr-Mo-Mn steel during deformation, it cannot describe the changes in the microstructure of the material and accurately predict its elastic deformation stage. In contrast, the Laasraoui segmented model can not only accurately predict the behavior of materials at all hot deformation stages, but also consider and describe the DRX behavior during deformation, which has a certain physical significance.

### 3.4. Grain Size Prediction Model

The deformation temperature and strain rate can significantly affect the grain size and influence their properties. The strength reduces when the grain size is too large [46]. The grain size prediction model was established for predicting the evolution of grain size under hot deformation.

**Figure 16:**
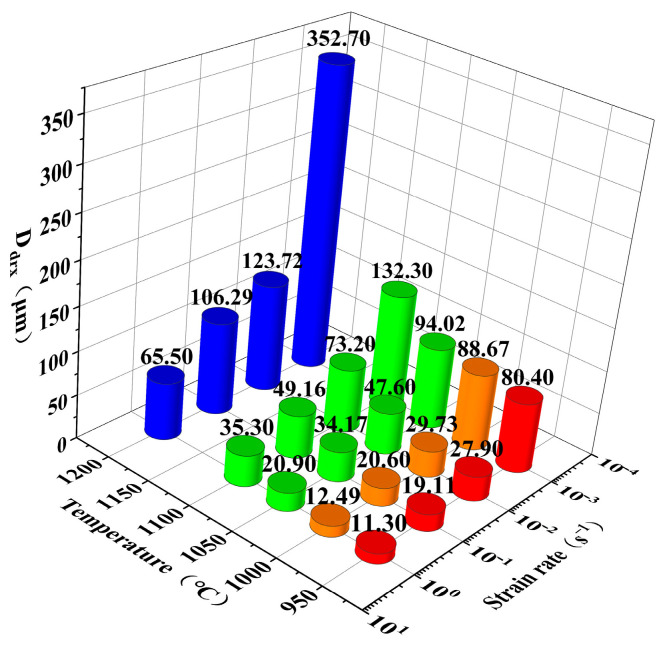
Ddrx under various deformation conditions.

**Figure 17:**
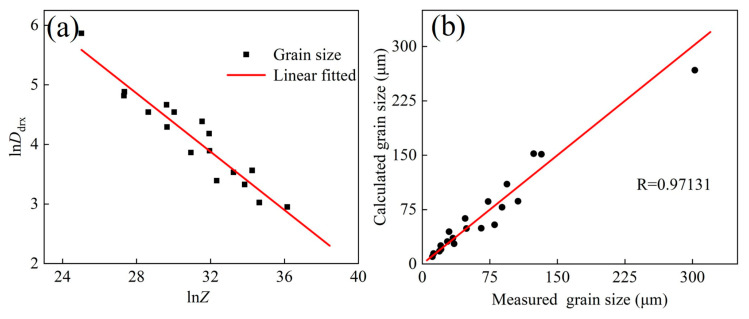
(**a**) Relationship between ln Ddrx and ln Z. (**b**) Fitted relationship between calculated and measured grain sizes.

Figure 16 shows the connection between DRX grain size (*D*_drx_), temperature, and strain rate. The *Z* parameter is also introduced in Figure 17a, and Formula (31) is obtained:

(31) $$D_{drx}=1.2243\times 10^{5}Z^{-0.2449}$$

Based on Formula (31), the average grain size under various deformation situations can be calculated, as shown in Formula (32):

(32) $$\bar{D}=D_{0}\left(1-X_{drx}\right)+D_{drx}X_{drx}$$

where $\bar{D}$ is the average grain size, and $D_{0}$ is the initial grain size; when $X_{drx}$ = 1, Formula (32) is equivalent to Formula (31).

As shown in Figure 17b, the calculated values of grain size are consistent with the experimental data, and its *R* is as high as 0.9713.

### 3.5. Construction of the HPMs

HPM is a powerful tool for optimizing the deformation process and revealing plastic deformation mechanisms [47]. The hot deformation of the Fe-Cr-Mo-Mn steel is considered as an energy dissipating body. The total power dissipation (*P*) consists of two components: the power dissipation caused by plastic deformation (G) and that caused by the internal microstructural evolution (J). The relationship between *P*, *J*, and *G* is expressed as follows:

(33) $$P=\sigma \overset{.}{\varepsilon }=\int_{0}^{\overset{.}{\varepsilon }}\sigma d\overset{.}{\varepsilon }+\int_{0}^{\sigma }\overset{.}{\varepsilon }d\sigma =G+J$$

When temperature remains constant, the correlations between σ and $\dot{\varepsilon }$ can be expressed using Formula (34).

(34) $$\sigma =k\cdot {\overset{.}{\varepsilon }}^{m}$$

where *k* is the material constant; *m* is the strain rate sensitivity exponent, which determines the distribution between *G* and *J* (Formula (35)); *J* is non-linearly correlated with strain rate and temperature. Formula (36) can be obtained by combining Formulas (33) and (34):

(35) $$m=\frac{\partial J}{\partial G}=\frac{\dot{\varepsilon }\partial \sigma }{\sigma \partial \dot{\varepsilon }}≅\frac{\partial ln\sigma }{\partial ln\dot{\varepsilon }}$$

(36) $$J=\int_{0}^{\sigma }\overset{.}{\varepsilon }d\sigma =\frac{m}{m+1}\dot{\varepsilon }\sigma$$

when *m* = 1, the material is considered to be in an ideal dissipative state, and *J* reaches its maximum.

The general power dissipation value (*η*) can be calculated by Formula (37) [48].

(37) $$\eta =\frac{J}{J_{max}}=\frac{2m}{m+1}$$

η depends on strain, strain rate, and temperature.

*η* represents the energy consumed by the microstructural evolution, such as DRV, DRX, and phase transition. The power dissipation value *η* > 35% can be expressed as a high-power dissipation region, which contributes to the hot processing of materials [49,50,51]. It should be noted that abnormal coarsening and flow instability may occur in the high-power dissipation region [52]. To evaluate the instability of the material, Formula (38) can be used [53]:

(38) $$\xi =\left[\frac{\partial \ln\left(m/m+1\right)}{\partial ln\dot{\varepsilon }}\right]+m\leq 0$$

where ξ represents the flow instability coefficient. Generally, the region ξ < 0 is considered to be the flow instability region.

#### 3.5.1. Power Dissipation Efficiency Maps

**Figure 18:**
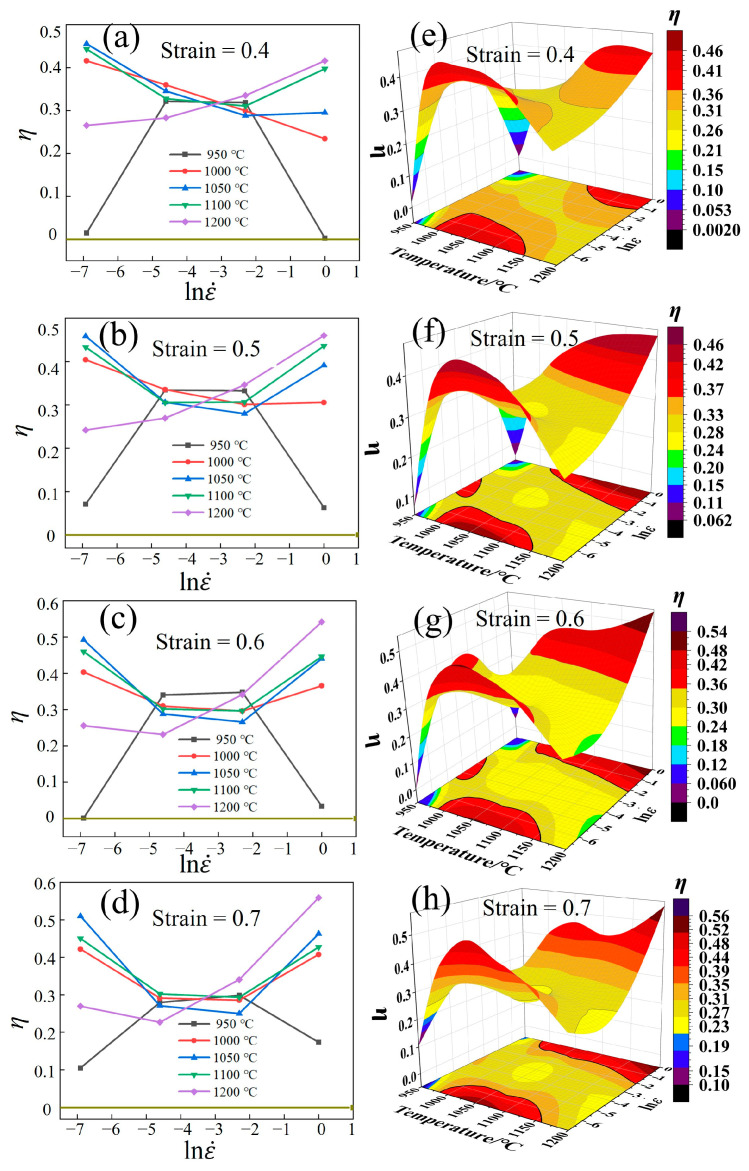
(**a**–**d**) Relationship between ƞ and strain rate. (**e**–**h**) Relationship between *ƞ* and strain rate for 3D maps of the Fe-Cr-Mo-Mn steel.

Figure 18a–d shows the relationship between *η*, strain rate, and temperature. At 950 °C, *η* initially rises and then declines as the strain rate increases. Under low strain rates, the hot deformation could consume more additional deformation energy, resulting in a lower *η*. At this time, complete DRX and grain coarsening are prone to occur. When *η* approaches 0, it is prone to form flow instability regions [37].

Under the temperature of 1000–1200 °C, *η* initially declines and then rises as the strain rate increases. The results demonstrate that the increase in the strain rate can promote the occurrence of DRX, reducing the additional energy consumption for the resistance of deformation, which enhances the plasticity and strength. This phenomenon is more significant at 1200 °C.

Figure 18e–h show 3D power dissipation efficiency maps, where the red and yellow regions represent the high-power and low-power dissipation regions. The red region is higher for *η* ≥ 35% regions. Chen et al. [54] suggested that the region where the power dissipation efficiency value is ≥35% can be considered as a high-power dissipation efficiency region. These regions are primarily found at high temperatures and low strain rates, such as 1070–1150 °C/0.001–0.0041 s^−1^ and 1050–1200 °C/0.67–1 s^−1^.

Higher deformation temperatures can promote GB migration, while lower strain rates extend the deformation time, thereby promoting DRX. The high-power dissipation regions are significantly expanded as the strain increases from 0.4 to 0.6, owing to the fact that the higher the degree of deformation, the more the DRX. The high-power dissipation region does not change significantly as the strain increases because the grains undergo complete DRX, and the dissipated power remains stable. The blue, brown, and black regions indicate the flow instability region within the temperature range of 950–970 °C and strain rates ranging from 0.001 s^−1^ to 0.002 s^−1^, and 0.367 s^−1^ to 1 s^−1^ (Figure 18e–h).

#### 3.5.2. Instability Maps

**Figure 19:**
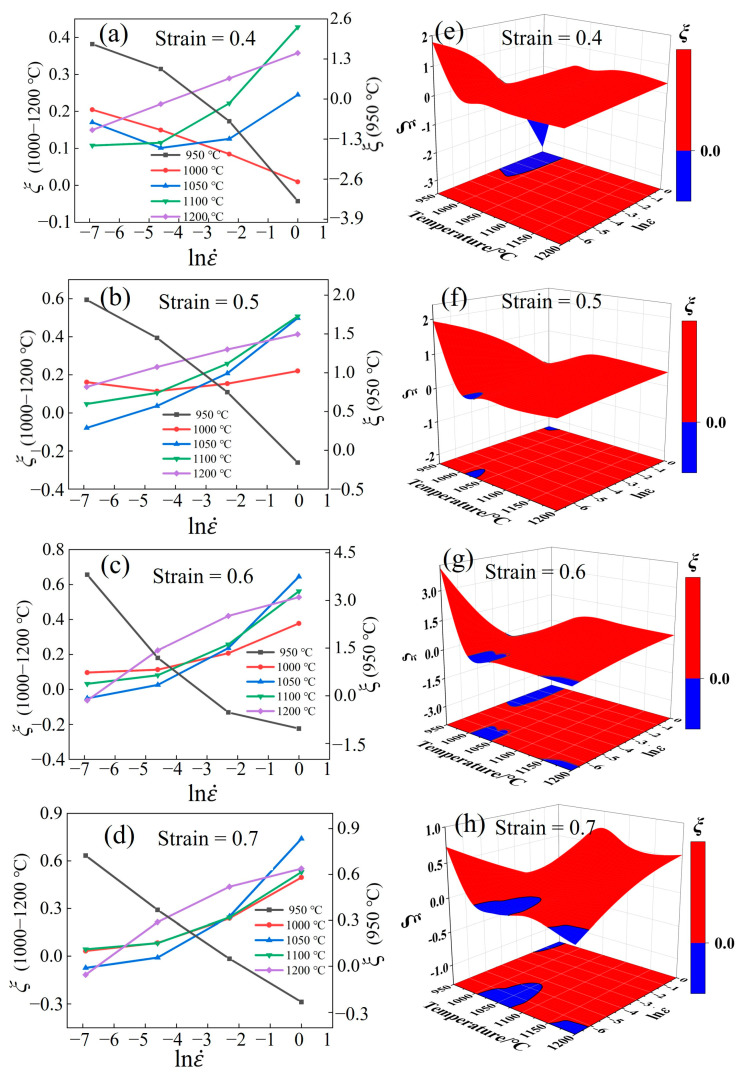
(**a**–**d**) Relationship between *ξ* and strain rate. (**e**–**h**) Relationship between *ξ* and strain rate for 3D maps of Fe-Cr-Mo-Mn steel.

Figure 19a–d show the curves of *ξ* varying with strain rate and temperature under different strains. The positive value of *ξ* is observed at 950 °C/0.001–0.1 s^−1^, as the extended duration of the low strain rate facilitates microstructural evolution processes such as DRV and DRX [55]. The *ξ* gradually becomes negative with the increase in the strain rate. Increased deformation accelerates the dislocation diffusion, and internal stress can easily result in microcracking and flow instability [56]. Furthermore, the increase in deformation also influences *ξ* at the same strain rate and temperature. For instance, when the processing was conducted at a strain of 0.4 under 1000 °C/1 s^−1^, *ξ* < 0. As the strain increased, *ξ* > 0. This is because large amounts of deformation can extend the time required for DRX. When the temperature exceeds 1000 °C, the unstable concentration region decreases and eventually disappears with increasing strain and strain rate.

Figure 19e–h shows the 3D instability maps. The blue area indicates the instability regions (*ξ* < 0), which should be avoided during hot deformation [57]. These regions are mainly concentrated in three regions: 1005–1045 °C/0.001–0.0041 s^−1^, 950–980 °C/0.05–1 s^−1^, and 1200 °C/0.001–0.0016 s^−1^.

#### 3.5.3. Analysis of HPMs

**Figure 20:**
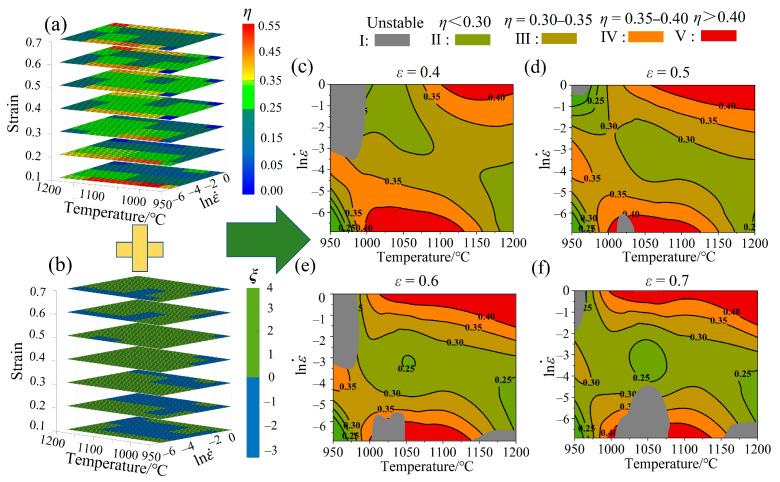
(**a**) 3D power dissipation efficiency maps. (**b**) 3D instability maps. (**c**–**f**) Processing maps under the true strain of 0.4–0.7.

Figure 20a,b show the power dissipation efficiency maps and instability maps of the Fe-Cr-Mo-Mn steel under the strain of 0.1–0.7. When the strain is less than 0.3, the instability region is large, and the hot deformation behavior just begins; its microstructure is relatively unstable, which is not representative of the range of hot processing. The Fe-Cr-Mo-Mn steel is widely used in large-scale steel forging, which requires high deformation during hot working. Therefore, a strain of 0.4–0.7 is usually selected in studies to obtain an optimal processing range.

Based on these, the HPMs can be obtained (Figure 20c–f). The HPMs can be divided into several regions based on *ξ.* The gray region I represents the instability regions (*ξ* < 0), while the processable regions (*ξ* > 0) can be further divided into four regions, namely, II (*η* < 30%, green), III (30% < *η* < 35%, yellow-green), IV (35% < *η* < 40%, orange), and V (*η* > 40%, orange). Among these, II and III are low-power dissipation regions, namely, the general processing regions, which are always along the orderly martensite packet and are beneficial for improving the hot processing performance of the material; however, they always undergo incomplete DRX, which is not conducive for the hot processing of the material. Regions IV and V exhibit high-power dissipation and are the optimal processing regions.

The DRX region increases alone with the high-power *η* region with the increase in strain (>0.4). Finally, the high-power *η* region gradually stabilizes with full DRX. According to Figure 20, the IV and V regions are formed under 1050–1200 °C/0.369–1 s^−1^ and 1050–1125 °C/0.001–0.0037 s^−1^. However, the latter process often results in instability. Considering actual production conditions, the 1050–1200 °C/0.369–1 s^−1^ range was selected as the optimal range of hot processing.

### 3.6. Analysis and Discussion

#### 3.6.1. Effect of Hot Deformation on High-Temperature Microstructure

**Figure 21:**
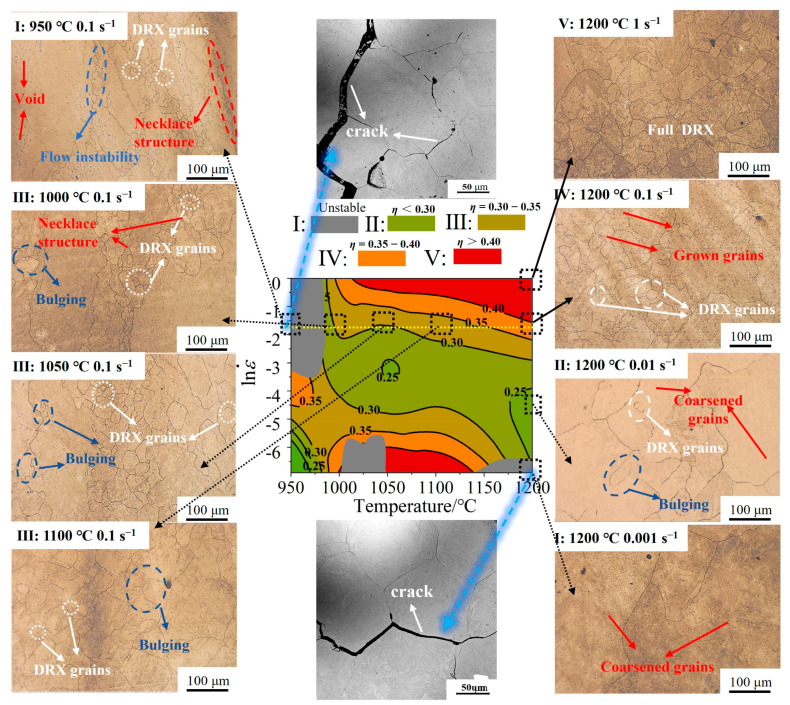
Microstructural characterization of the regions of HPM under a strain of 0.6: (I) microcrack and flow instability, (II–IV) grain growth, (V) optimal processing region.

To validate the accuracy of the processing maps, the microstructure of the Fe-Cr-Mo-Mn steel under various hot deformation conditions at a strain of 0.6 was characterized, as shown in Figure 21. Combined with Figure 20e, microcracks were seen to have formed in the I region, which appeared between two coarse crystals. In the II–III regions, the microstructure exhibited incomplete DRX grains. As the temperature increased, the proportion of DRX increased, which is consistent with the model in Figure 12a. The microstructure of the mixed grains diminished as DRX increased due to the promotion of DRX. The serrated bending of the GBs was observed at a temperature of 950 °C, accompanied by the occurrence of typical flow instability at the interface between coarse and fine grains. Additionally, smaller DRX grains were generated around larger grains, resulting in the formation of a necklace structure. The GBs of large grain sizes gradually exhibited conspicuous bulging with the increase in temperature, which is commonly regarded as a precursor of DRX nucleation. When the temperature increased to 1200 °C, the bulging GBs were replaced by equiaxed DRX grains. The migration of GBs was facilitated at a high temperature (1200 °C), resulting in the formation of coarse grains. With the same strain rate, *η* gradually increased, which was beneficial to hot workability as the temperature rose. In the regions of I–V, the strain rate was 0.001–1 s^−1^, and the microstructure was complete DRX. The grain size decreased with the increase in the strain rate. When the strain rate was 0.001 s^−1^, the grains were partially coarsened. When the strain rate was 0.01 s^−1^, equiaxed, bulging, and coarse-grained mixed grains appeared, which have an adverse effect on isothermal die forging. At a strain rate of 1 s^−1^, equiaxed crystal grains appeared. At this time, DRX grains were uniformly nucleated, and austenite grains do not grow abnormally under the coupling effect of temperature and stress. This is similar to the work of Chen et al. [54] on HPMs of an alloy steel; they found that equiaxial DRX grains in the optimal processing region are conducive to isothermal die forging.

#### 3.6.2. Effect of Hot Deformation Behavior on Quenched Structure

**Figure 22:**
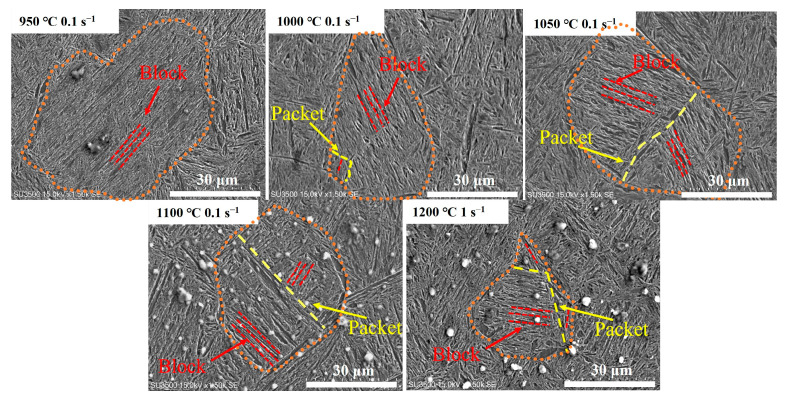
Quenched structure under different deformation conditions by SU3500 SEM.

Martensitic transformation has an important effect on the mechanical properties of the steel. Figure 22 shows the quenched structure under different hot deformation conditions, where the orange dotted line is the austenite GB, the yellow dotted line is the martensitic packet GB, and the red dotted line is the martensitic block GBs. When the deformation condition was 950 °C/0.1 s^−1^, the martensite block was regularly ordered within the austenite boundaries while randomly arranged in the outer regions of the boundaries without any martensite packet. The DRX grains were relatively small, and it is difficult to observe the austenitic GBs of other DRX grains. The current state of the martensite structure was disordered, lacking any identifiable multistage martensite. As the temperature increased under (1000–1200 °C)/0.1 s^−1^, the multistage martensite appeared, in which the enveloped martensite composed of block-shaped martensite formed in the boundary of austenite. When the deformation condition was 1200 °C/1 s^−1^, the martensite packet within the austenite GB increased, and the transformation of multistage martensite was more significant.

With the increase in temperature and strain rate, high strain was generated locally, which increased the energy loss of the material and promoted martensitic transformation. The phase transition interface of multistage martensite could act as the preferred nucleation site for DRX grains, which has a certain promoting effect on the nucleation and growth of DRX. As the degree of DRX increased, more multistage martensite formed, accompanied by the ordering of block martensite.

**Figure 23:**
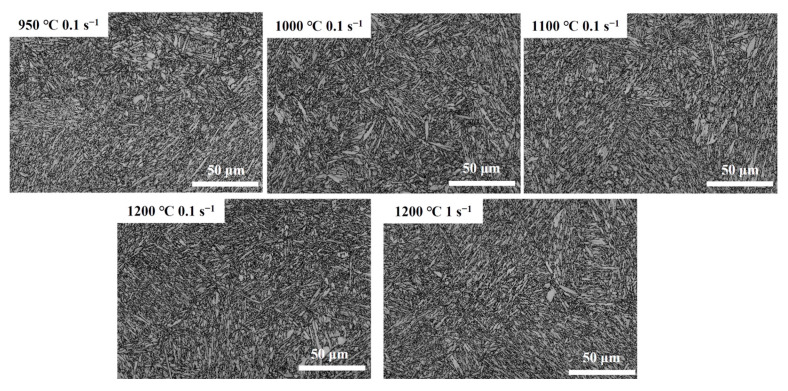
BC plots of the Fe-Cr-Mo-Mn steel at different deformation conditions.

**Figure 24:**
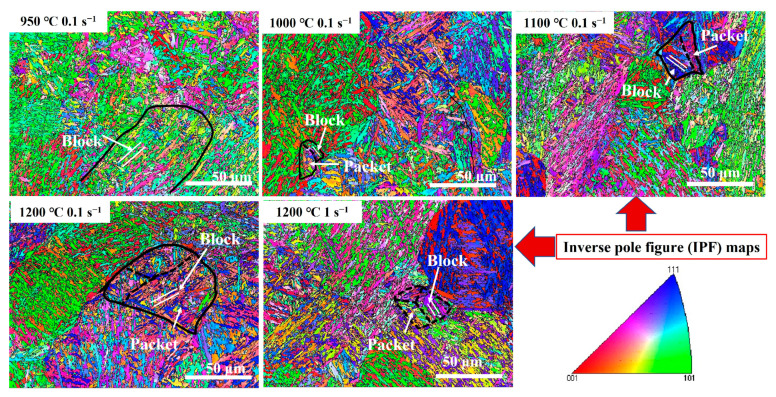
IPF post-diagram of the Fe-Cr-Mo-Mn steel under different deformation conditions.

**Figure 25:**
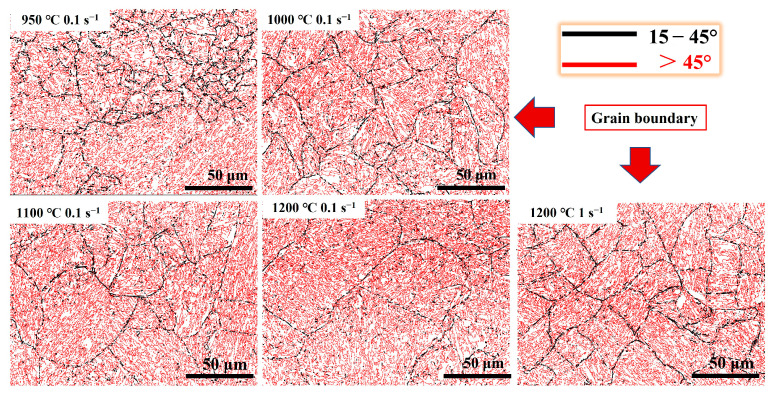
GBs post-diagram of the Fe-Cr-Mo-Mn steel under different deformation conditions.

Figure 23, Figure 24 and Figure 25 show the band comparison (BC), inverse pole figure (IPF), and GB of the quenched Fe-Cr-Mo-Mn steel. As shown in Figure 23, the quenched structure of the Fe-Cr-Mo-Mn steel is mainly lath martensite. As shown in Figure 24, with the increase in the deformation temperature and strain rate, the martensite packet gradually appeared, and the multistage structure of the martensite appeared; at 1200 °C, the martensite packet was found within GBs of austenite, and there were regularly arranged martensite blocks inside. This is consistent with the phenomenon found in Figure 22. In Figure 25, the black line is the medium angle grain boundary (MAGB = 15–45°), and the red line is the high angle grain boundary (HAGB > 45°). The intermediate angle grain boundary gradually decreased with the increase in temperature and decrease in strain rate.

**Figure 26:**
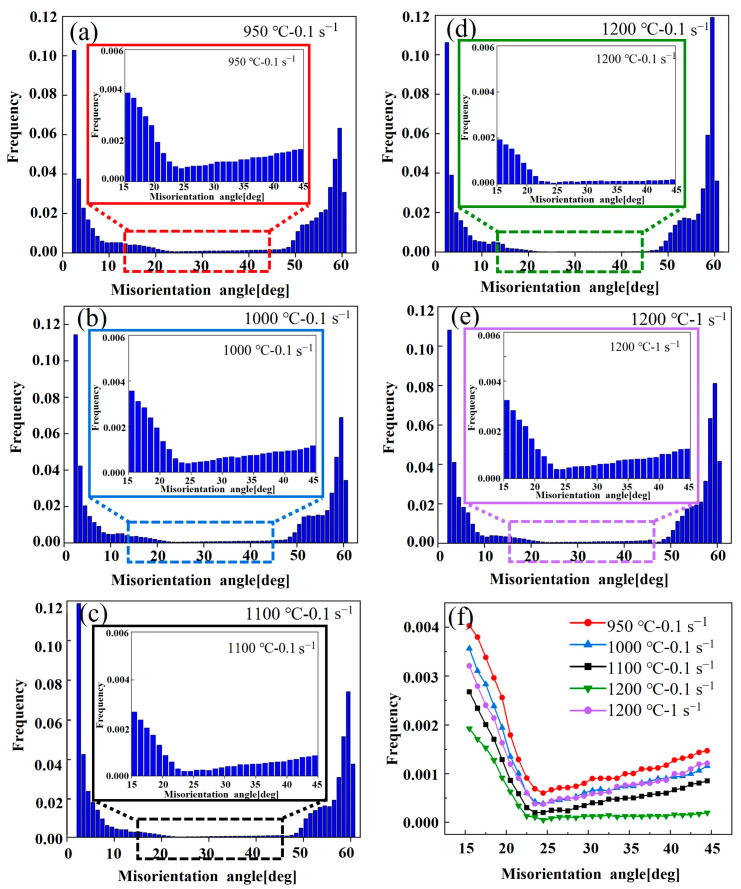
Misorientation angle distributions of the Fe-Cr-Mo-Mn steel under different deformation conditions: (**a**) 950 °C-0.1 s^−1^, (**b**) 1000 °C-0.1 s^−1^, (**c**) 1100 °C-0.1 s^−1^, (**d**) 1200 °C-0.1 s^−1^, (**e**) 1200 °C-1 s^−1^, (**f**) comparisons of middle misorientation angle distributions.

The lath martensite with a low angle grain boundary (LAGB < 15°) dominates the quenched structure under each hot deformation condition (Figure 26). The MAGB is mainly derived from deformed austenite [58]. As the temperature decreased and the strain rate increased, the MAGB fraction gradually increased, indicating that more substructures formed in the initial austenite. Wang et al. [33] demonstrated through an investigation of 42CrMo that a higher MAGBs frequency indicates that the initial austenite deforms to form more substructures at lower temperatures. This means that the substructures introduced by austenite deformation could hinder the formation of multiphase martensite.

**Figure 27:**
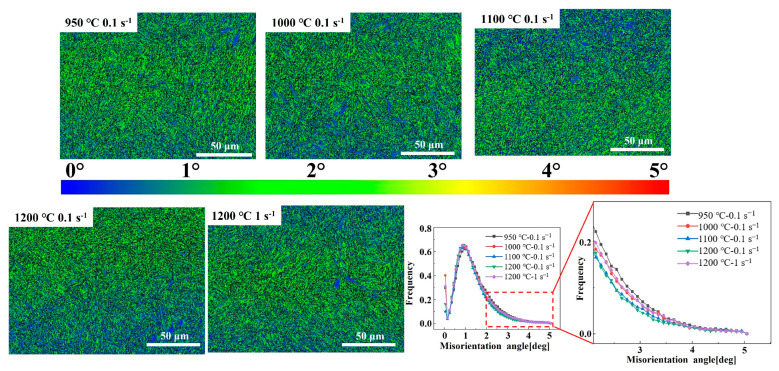
Local misorientation maps of the Fe-Cr-Mo-Mn steel under different deformation conditions.

Figure 27 is the kernel average misorientation (KAM) of the quenched Fe-Cr-Mo-Mn steel. Locations with higher values indicate a greater degree of plastic deformation or higher defect density. The quenched structure at low deformation temperatures exhibits a high local dislocation, while the KAM value gradually increases as the strain rate increases and the deformation temperature decreases. When DRX occurs, most dislocations are consumed, and the dislocations introduced by phase transitions and substructures inherited from austenite deformation can be preserved. Li et al. [59] demonstrated that the occurrence of DRX can effectively reduce the dislocation density and deformation energy during the deformation process.

When DRX was incomplete, the proportion of MAGB and average local strain were high in the quenched sample, and the multistage martensite could be destroyed by a higher-density dislocation. When DRX was complete, a typical multistage martensite tended to form. It was also demonstrated that the residual substructure in the deformed austenite can be preserved at the quenched structure by a phase transformation. Furthermore, this residual substructure also hinders the formation of multistage martensite.

The complete DRX and multistage structures of the martensitic collaborate in regions IV and V. The DRX could enhance the plastic deformation and forming ability of the material. The presence of a multistage martensitic structure could act as strong obstacles to the dislocation motion and refine the grain size, increasing the strength and hardness of the Fe-Cr-Mo-Mn steel. Xu et al. found that the multistage martensite can be induced by stress [60]. The formation of this phase could further reduce the stress. Therefore, the mechanical strength and plasticity are both considerable under the mutually effects between DRX and multistage martensite.

## 4. Upsetting Experiment of Imitation Hammer Shaped Parts

### 4.1. Simulation Model

We modified the user-defined material stress routine (USRMTR) in the source file usr_mtr.f with Fortran language; material stress was defined as strain, strain rate, and user cell/node variables. The UFLOW number was called by the USRMTR during pre-processing and returned to the superior program. The Laasraoui segmented flow stress model presented in this paper was written in this source file. The DRX and grain size model in this paper were modified in another source document usr_upd.f. The user-pre-defined unit/node variable program (USRUPD) was programmed to calculate the initial value with USRE and the resulting value with USRN. The user-defined variables related to the secondary development in this paper and their implications are shown in Table 6.

**Figure 28:**
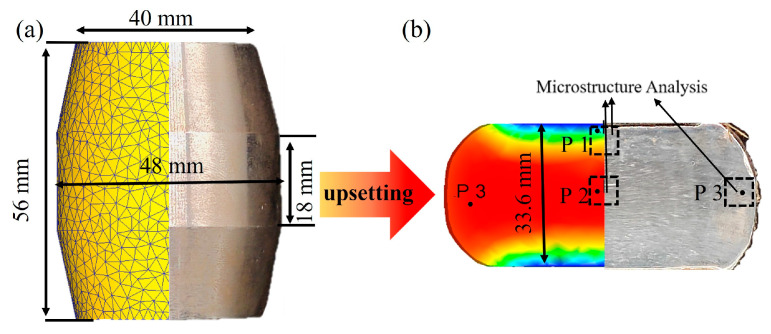
Modeled and experimental workpieces and experimental and simulated workpieces after upsetting: (**a**) initial workpieces, (**b**) Deformed workpieces.

The workpiece is shown in Figure 28. The top and bottom dies were made of H13 die steel. H13 die steel has high hardness, and when set at room temperature 25 °C, its deformation during hot compression can be negligible. Theoretically, the hot compression experiment is carried out under a fixed deformation temperature and strain rate. In fact, the temperature field of the sample in the actual compression deformation is instantaneous because the material plastic deformation work is transformed into heat energy in the deformation process, and the heat transfer, convection, and radiation occur between the sample and the surrounding environment. At the same time, the instantaneous strain rate of each point inside the sample also changes constantly. Due to the small size of the sample and the short compression time, the temperature field does not change much, so the simplification is made here. The temperature field of the sample is considered to remain unchanged during the simulation process, and the heat transfer between the sample and the environment and the anvil are ignored, so the anvil is selected as rigid in the numerical simulation to eliminate the influence on the numerical simulation calculation of the workpiece.

**Figure 29:**
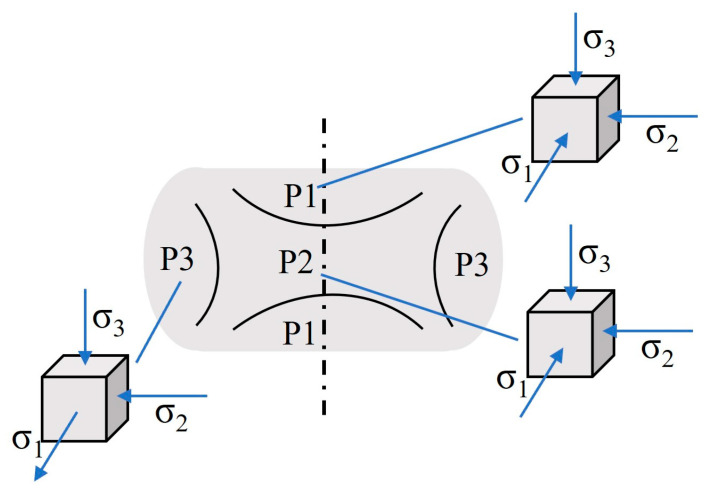
Schematic diagram of the partition and stress state of the compressed sample after deformation.

The starting temperatures of the anvil and workpiece were respectively set to 25 °C and 1150 °C. Between the workpiece and the air, the heat exchange coefficient was 0.02 N/(mm·s·°C); between the dies, the heat exchange coefficient was 11 N/(m·s·°C), and the friction factor was 0.5. The unit type of the workpiece was a tetrahedral mesh, the number of elements was 17,365, and the upper die pressing speed was 15 mm/sec; the deformation of the workpiece was 40%. According to the principle of pressure machining, the external friction during compression is the friction generated when the friction between the anvil and the specimen end face are in contact (σ_3_). With the increase in deformation in the compression process, the friction between the mold and the sample increase. The friction force changes the flow characteristics of the metal during compression deformation, which directly affects the stress and strain distribution of the material. The deformation of the workpiece is divided into three parts, as shown in Figure 29: the upper and lower difficult deformation region (P1), the central large deformation region (P2), and the side small deformation region (P3). In region P1, σ_3_ causes transverse pressures σ_1_ and σ_2_. Compared with regions P2 and P3, the stress states of region P1 are hardly meet the plastic conditions and have the least influence on the flow behavior. From the vertical direction, the region P1 is the most affected by friction, and the region P2 is the least affected by friction. But the P2 region is in the center, the metal flow is hindered by the outer, the deformation resistance is the greatest, and the flow behavior is the most affected.

### 4.2. Numerical Simulation

**Figure 30:**
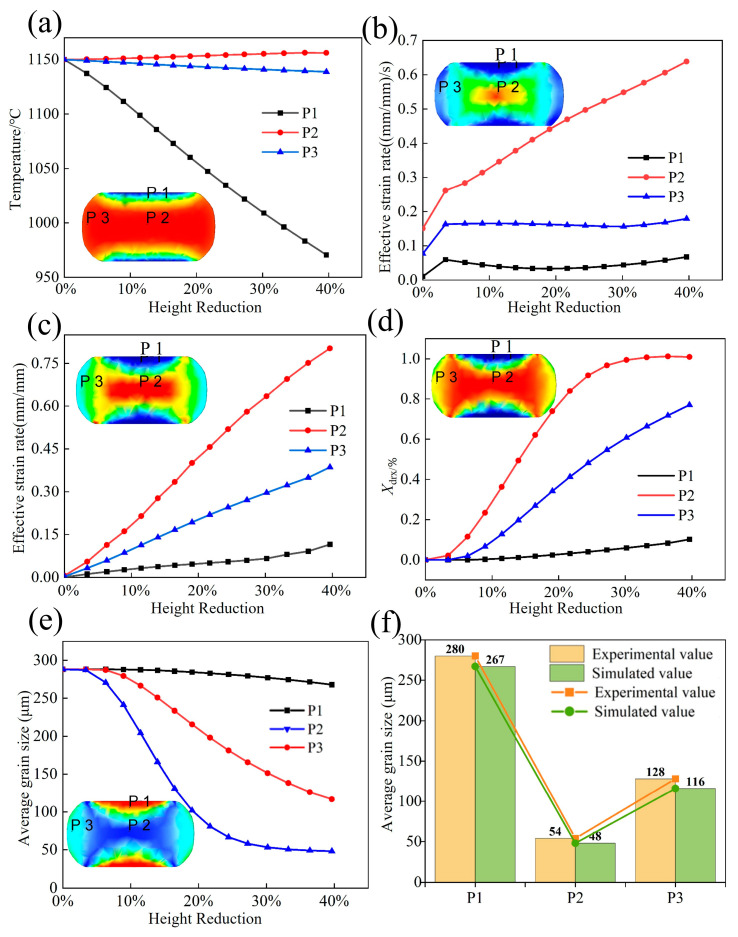
Comparison of analog values at P1, P2, and P3 points. (**a**) Temperature, (**b**) Effective strain rate, (**c**) Effective strain, (**d**) DRX percentage, (**e**) Average grain size, (**f**) Comparison of simulated and experimental average grain sizes.

The numerical simulation results of P1, P2, and P3 regions are shown in Figure 30. As shown in Figure 30a, since point P1 is located at the contact position between the workpiece and the die, the temperature drops sharply to 960 °C, and the temperature in the area of P2 rises to 1156 °C due to the heat effect of plastic deformation. The temperature rises gradually from the surface layer to the center along the radial direction.

The attenuation of the strain rate could cause the deterioration of deformation uniformity and coordination [61], which easily leads to instability. Figure 30b shows the effective strain rate under different positions. The effective strain rate did not attenuate significantly, and the upsetting workpiece was in the best processing region, verifying the accuracy of the HPMs.

Effective strain is an important index for evaluating the penetration of forged parts [61]. The effective strain changes under different positions of forged parts are shown in Figure 30c. The P1 point was greatly affected by the end-face friction, and the local effective effect became 0.124. There was a very small deformation. The P2 point was the least affected by the outer end friction, and the local effective strain reached the maximum of 0.82. The P3 point was greatly affected by the circumferential tensile stress of the end face, so the strain was less than P2, and the effective strain became 0.41. During the upsetting process, the workpiece was affected by external friction, so that the equivalent strain was unevenly distributed. The large strain in the heart of the forgings contributes to enhancing the microstructural properties. The average grain size in different regions was compared with the simulation results, and the fitting accuracy of the experimental and simulated values was high, as shown in Figure 30f.

### 4.3. Result Analysis

**Figure 31:**
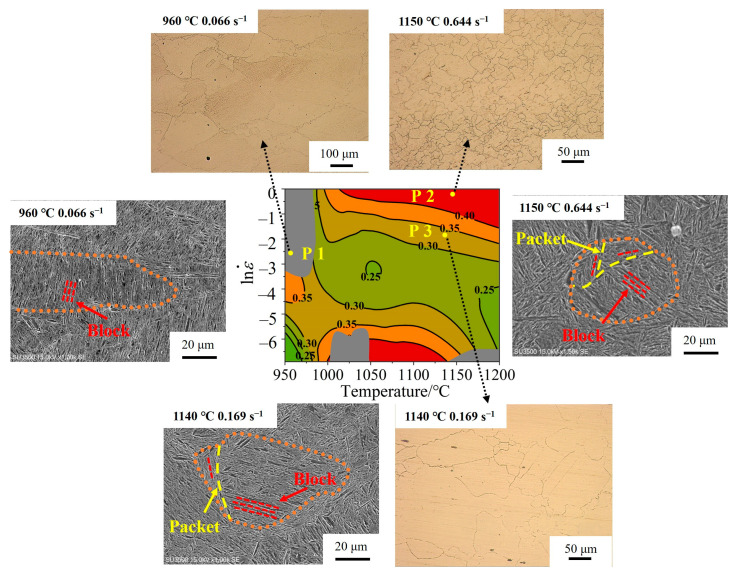
P1, P2, and P3 points in the HPM and the microstructure of each point.

The HPM with a strain of 0.6 was selected as the final verification. The microstructural features of P1, P2, and P3 points are shown in Figure 31, while their positions within the processing map were determined based on the simulated results in Figure 30.

The P1 point (960 °C/0.066 s^−1^) was processed in the unstable region, and the flow instability occurred. Serrated GBs were found without any multistage martensite. The *X*_drx_ was about 10% (Figure 30d), and DRX grains were hardly observed, which is consistent with the characteristics of the instability region. The *X*_drx_ of point P2 (1150 °C/0.644 s^−1^) reached 100%. Fine DRX equiaxed grains were formed, and the grain size was reduced six times compared to the initial grain size. The multistage martensite was formed, which is consistent with the characteristics of the optimum processing region. The *X*_drx_ of P3 point (1140 °C/0.169 s^−1^) was 75%, which presented incomplete DRX characteristics, and the multistage martensitic began to appear.

## 5. Conclusions

(1) As the deformation temperature decreased or the strain rate increased, the grain size and *X*_drx_ of the Fe-Cr-Mo-Mn steel gradually decreased, while the flow stress gradually increased. Highly precise Laasraoui segmented and Arrhenius models were established, and the correlation coefficient *R* of each was as high at 0.99784 and 0.99679. Moreover, DRX and average grain size models were developed.
(2) The substructure introduced by the austenite deformation could inhibit the formation of multi-stage martensite. With the increase in temperature or strain rate, the structure of martensite evolved from single-stage to multistage with order transformation. In the optimum processing region, the mixture microstructure composed by complete DRX and multistage martensitic enhance the hot deformation capability of the material.
(3) The optimal processing parameters for the Fe-Cr-Mo-Mn steel were determined: a temperature range of 1050–1200 °C and a strain rate range of 0.369–1 s^−1^. The secondary development Laasraoui segmented model and the microstructure evolution model were embedded into the Deform-3D subroutine to verify the result of the upsetting experiment. The accuracy of the models was demonstrated, providing a robust theoretical foundation for the hot forging processes of the Fe-Cr-Mo-Mn steel.

## Figures and Tables

**Table 1:** Chemical composition of Fe-Cr-Mo-Mn steel (%, mass fraction).

C	Si	Mn	P	S	Cr	Mo	Cu	Fe
0.41	0.26	0.69	0.0063	0.0091	1.11	0.20	0.03	Bal.

**Table 2:** *B*_p_ of Fe-Cr-Mo-Mn steel at different hot deformation conditions.

Deformation Temperature (°C)	Strain Rate (s^−1^)			
	0.001	0.01	0.1	1
950 °C	1.0321	0.9691	1.0698	1.0232
1000 °C	1.0926	1.0497	0.9841	1.0677
1050 °C	1.0527	1.0423	1.0598	1.0698
1100 °C	0.9941	1.0432	1.0872	1.0921
1200 °C	1.0863	1.0818	1.0734	1.0310

**Table 3:** Model parameter values for different strains.

Strain	*α*	*n*	*Q*/(kJ/mol)	ln*A*
0.10	0.021899869	4.587893	407.311	31.69569
0.15	0.020026631	4.346701	408.382	31.81251
0.20	0.019169447	4.101312	397.747	30.94919
0.25	0.018783997	3.932011	388.867	30.27511
0.30	0.018665743	3.814459	383.540	29.90283
0.35	0.018649029	3.795894	384.477	30.07963
0.40	0.018797260	3.767556	382.181	29.94070
0.45	0.018938808	3.747479	381.805	29.94879
0.50	0.019037187	3.800027	384.209	30.20930
0.55	0.019212378	3.814247	385.030	30.31099
0.60	0.019341395	3.823353	389.404	30.71907
0.65	0.019442589	3.828529	392.002	30.95220
0.70	0.019472766	3.862982	394.066	31.14102

**Table 4:** Results of polynomial fitting of *α*, *n*, *Q*, and ln*A* to the true strain.

*α*		*n*		*Q*		ln*A*	
*X*_0_	0.03018	*N*_0_	5.01577	Q_0_	373,753	Y_0_	29.10331
*X*_1_	−0.12723	*N*_1_	−2.17646	Q_1_	795,498.55	Y_1_	62.23573
*X*_2_	0.54696	*N*_2_	−32.96995	Q_2_	−5,997,871.49	Y_2_	−474.19401
*X*_3_	−1.15679	*N*_3_	143.72186	Q_3_	17,025,652.65	Y_3_	1363.84008
*X*_4_	1.2227	*N*_4_	−210.73112	Q_4_	−21,094,491.99	Y_4_	−1703.96763
*X*_5_	−0.51441	*N*_5_	106.06187	Q_5_	9,686,499.7	Y_5_	786.60111

**Table 5:** The values of the various parameters.

Temperature/°C	$\overset{.}{\varepsilon }$	$\sigma_{c}$/MPa	$\sigma_{p}$/MPa	$\sigma_{s}$/MPa	$\sigma_{ss}$/MPa	$\varepsilon_{c}$	$\varepsilon_{p}$
950	0.001	42.37	58.93	73.56	58.2	0.091	0.221
	0.01	65.69	80.81	88.29	64.62	0.076	0.204
	0.1	85.44	116.49	122.95	110.45	0.073	0.320
	1	115.47	147.73	155.97	147.23	0.098	0.441
1000	0.001	27.82	37.13	42.82	32.40	0.058	0.189
	0.01	54.61	67.16	80.85	53.03	0.066	0.201
	0.1	70.42	94.26	98.24	75.78	0.073	0.278
	1	101.39	127.56	140.58	122.15	0.096	0.369
1050	0.001	23.89	30.49	38.07	26.30	0.051	0.136
	0.01	47.87	56.32	67.14	44.12	0.064	0.151
	0.1	62.91	81.13	88.97	60.87	0.070	0.212
	1	79.47	107.52	113.77	102.70	0.075	0.352
1100	0.001	18.88	23.82	28.68	20.95	0.049	0.131
	0.01	39.43	45.05	48.99	34.75	0.065	0.133
	0.1	54.50	68.58	72.75	50.81	0.069	0.205
	1	69.29	94.06	103.77	82.62	0.069	0.329
1200	0.001	13.62	15.45	17.67	14.32	0.048	0.112
	0.01	19.23	22.10	30.42	19.10	0.059	0.159
	0.1	29.73	38.52	54.94	28.12	0.065	0.215
	1	40.66	54.39	62.39	54.15	0.097	0.316

**Table 6:** Implications of user-defined variables.

User Variables	USRE (1)	USRE (2)	USRE (3)	USRE (4)	USRE (5)	USRE (6)	USRE (7)
Implication	ε-Equivalent	*Z*	$\varepsilon_{p}$	$\varepsilon_{c}$	X_drx_	D_drx_	$\bar{D}$

## Data Availability

The data that support the findings of this study are available from the corresponding author upon reasonable request.
